# *TFAP2E* Expression Is Associated with Survival and a Transcriptomic Pattern in Ovarian Serous Carcinoma, Supported by Hallmark and Protein-Level Characterization

**DOI:** 10.3390/cancers18193168

**Published:** 2026-10-01

**Authors:** Damian Kołat, Julia Gałęziewska, Paulina Buczek, Lin-Yong Zhao, Mateusz Kciuk, Żaneta Kałuzińska-Kołat, Małgorzata Kozłowska, Agnieszka Śliwińska, Renata Kontek, Elżbieta Płuciennik

**Affiliations:** 1 Department of Functional Genomics, Medical University of Lodz, 90-752 Lodz, Poland; julia.galeziewska@umed.lodz.pl (J.G.); paulina.buczek@stud.umed.lodz.pl (P.B.); mateusz.kciuk@biol.uni.lodz.pl (M.K.); zaneta.kaluzinska@umed.lodz.pl (Ż.K.-K.); elzbieta.pluciennik@umed.lodz.pl (E.P.); 2 Department of Molecular Biotechnology and Genetics, University of Lodz, 90-237 Lodz, Poland; renata.kontek@biol.uni.lodz.pl; 3 International Doctoral School, Medical University of Lodz, Hallera Square 1, 90-647 Lodz, Poland; 4 Department of General Surgery, West China Hospital, Sichuan University, Chengdu 610041, China; 153795352@scu.edu.cn; 5 Department of Nucleic Acid Biochemistry, Medical University of Lodz, 92-213 Lodz, Poland; malgorzata.kozlowska@umed.lodz.pl (M.K.); agnieszka.sliwinska@umed.lodz.pl (A.Ś.)

**Keywords:** *TFAP2E*, ovarian serous carcinoma, transcriptomic signature, biomarkers, bioinformatics, survival analysis, expression profiling, weighted gene correlation network analysis, cancer hallmarks, immunohistochemistry

## Abstract

**Background/Objectives:** AP-2 transcription factors are increasingly recognized as key regulators in cancer biology. The *TFAP2E* gene, which encodes the AP-2ε transcription factor, remains understudied in cancer compared with other AP-2 family members. The scarcity of studies examining potential AP-2ε targets and related genes limits our understanding of its functional significance. Moreover, related studies have not incorporated a screening step to select the tumor type for justified molecular investigation. Through comparison of tumor–normal expression differences in a pan-cancer spectrum, this study prioritized ovarian serous carcinoma for downstream assessment. **Methods:** Expression patterns were evaluated using GENT2, GEPIA2, and TNMplot. After prioritizing ovarian serous carcinoma, *TFAP2E* and related signatures in TCGA-OV were analyzed using transcriptomic and clinical data from GDC and CDR, followed by comparison of findings with the GSE32062 cohort. The workflow included survival and clinicopathological analyses, outcome-independent limma-voom differential expression analysis and WGCNA, MCODE clustering, gene ontology, immune subtype and stemness assessment, PCA, CancerHallmarks enrichment, clinically adjusted Cox and competing-risk survival modeling, and HPA-derived immunostaining evaluation. Additional analyses assessed WGCNA parameter stability and preservation in GSE32062, an outcome-independent MAD-based variability screen, AP-2 family coexpression, and promoter motif enrichment. **Results:** *TFAP2E* was downregulated in ovarian cancer across the three queried resources, whereas higher expression was associated with favorable survival across multiple endpoints. Differential expression and WGCNA identified a *TFAP2E*-associated signature; its ontology indicated GPCR, stimulus responsiveness, and KRAS enrichment for the 933-gene module. The WGCNA pink module was stable across alternative settings, moderately preserved in GSE32062 (Zsummary = 7.86), and enriched in the outcome-independent MAD screening. GSE32062 supported concordant gene expression patterns and showed an association of *TFAP2E* with progression-free survival. Exploratory immune analyses showed a modestly increased proportion of C5 tumors in the higher-expression groups, whereas differences in stemness were statistically insignificant. The *TFAP2E* group associations with PFI and DSS in TCGA-OV and with PFS in GSE32062 remained significant after clinical adjustment; Fine-Gray sensitivity analyses gave consistent results for DSS. Immunohistochemistry provided protein-level characterization; eighteen entries differed between representative normal and tumor specimens, with lower tumor staining in fifteen. The seven-gene candidate set showed a clinically adjusted DSS association in TCGA-OV, which was not supported in the GSE32062 evaluation model. **Conclusions:** *TFAP2E* expression is associated with survival and a transcriptomic cross-cohort pattern in ovarian serous carcinoma. Covariate-adjusted and competing-risk analyses supported the association but did not establish clinical utility. The highlighted genes remain exploratory candidates for mechanistic investigation.

## Simple Summary

*TFAP2E*, encoding the AP-2ε transcription factor, remains poorly characterized in cancer despite the established regulatory importance of the AP-2 family. This study first evaluated tumor–normal expression differences across a pan-cancer spectrum to prioritize ovarian serous carcinoma for investigation, and then integrated transcriptomic, clinical, survival, network, pathway, multivariable, hallmark, and protein-level analyses. The GSE32062 cohort was used to assess the consistency of the main expression patterns, progression-related associations, and exploratory immune/stemness investigation. *TFAP2E* was consistently downregulated in ovarian tumors, whereas higher expression in cancer patients was associated with more favorable survival outcomes. Differential expression and correlation analyses identified a *TFAP2E*-associated pattern characterized by, e.g., GPCR- and KRAS-related ontological signals, accompanied by cancer hallmark and immunohistochemical characterization. Seven highlighted genes were retained as exploratory survival-associated candidates for further research. Collectively, the study identifies a distinct ovarian serous carcinoma transcriptomic pattern associated with *TFAP2E* expression and provides candidates for mechanistic and translational follow-up.

## 1. Introduction

The activating enhancer-binding protein 2 (AP-2) family belongs to the basic Helix–Span–Helix (bHSH) superclass of transcription factors (TFs) and comprises five members (AP-2α, AP-2β, AP-2γ, AP-2δ, AP-2ε) encoded by *TFAP2A-E* genes [1]. These proteins play crucial roles in regulating gene expression during embryonic development and differentiation, orchestrating processes such as proliferation, apoptosis, and cell cycle control [2]. The structural organization of AP-2 family members includes an amino-terminal transactivation domain and a carboxyl-terminal region responsible for DNA binding and dimerization [3]. When interacting with their target genes, all members recognize evolutionarily conserved motifs rich in guanine and cytosine, including GCCN_3/4_GGC, GCCN_3/4_GGG, or CCCCAGGC [4].

AP-2 transcription factors are increasingly recognized as context-dependent regulators in tumor biology. Altered functionality of these proteins has been shown to play crucial roles in cancer development and progression, with significant prognostic implications for oncological patients [5,6]. The activity of AP-2 family members in carcinogenesis is highly dependent on the specific member and tumor type [1], with the roles of AP-2α/β/γ described more thoroughly than the rest of the family [7,8,9,10]. AP-2δ and AP-2ε remain comparatively understudied, which may reflect their later discovery and the earlier presumption that they could share redundant functions with other AP-2 proteins. Limited studies suggest that AP-2δ may be associated with progression in prostate cancer and genome organization in lung cancer [11,12], while AP-2ε has been found to act as a tumor suppressor in neuroblastoma and colorectal cancer [13,14]. Although AP-2ε has accumulated compelling preliminary evidence of cancer-relevant functions, its downstream molecular context remains mostly undefined. The scarcity of studies collecting data on potential AP-2ε targets and related genes limits our understanding of its functional significance. Moreover, *TFAP2E*-focused studies have not incorporated a screening step to select the tumor type for justified molecular investigation.

In view of the presented data, a pan-cancer analysis of *TFAP2E* expression was first conducted to identify cancer types where this gene might play a significant role, followed by an exploratory workflow in the tumor with the most consistent signal. Downregulation of *TFAP2E* in ovarian tumors relative to normal ovarian tissue was revealed across multiple datasets. The prognostic significance of *TFAP2E* expression in ovarian serous carcinoma was further investigated, along with the transcriptomic processes associated with *TFAP2E* expression in this malignancy. Through comprehensive analyses of survival outcomes, expression profiles, gene ontologies, immune subtypes, stemness indices, and immunostaining data, our study characterizes the molecular and prognostic associations of *TFAP2E* expression in ovarian serous carcinoma and examines related genes as exploratory candidates for future mechanistic and clinical assessment.

## 2. Materials and Methods

### 2.1. Analysis of TFAP2E Gene Expression Patterns in a Pan-Cancer Spectrum

An assessment of *TFAP2E* gene expression differences between normal and tumor tissues was conducted across multiple cancer types, utilizing three databases: Gene Expression patterns across Normal and Tumor tissues v2 (GENT2), Gene Expression Profiling Interactive Analysis v2 (GEPIA2), and Tumor/Normal/Metastatic plot (TNMplot). GENT2 [15] was used to examine *TFAP2E* expression patterns across various tissues using microarray data through the “Search—Gene Profile” workflow. The GPL570 platform (Affymetrix Human Genome U133 Plus 2.0 Array) was used for tissue comparisons. Results were presented as boxplots, depicting median expression values and interquartile ranges, with statistical significance assessed using a two-sample *t*-test (*p* < 0.05). *TFAP2E* expression was also evaluated using the Single Gene Analysis function of GEPIA2 [16] by inputting the gene symbol. Expression profiles were visualized using dotplots with a log2 transformation. One-way analysis of variance (ANOVA) was applied to determine statistical significance (*p* < 0.05) of expression differences between tissue types. TNMplot [17] was employed via the “pan-cancer analysis” option to compare RNA sequencing (RNA-Seq) data concerning *TFAP2E*. Statistical significance was determined using the Mann–Whitney U test with a threshold of *p* < 0.05. Expression values were not pooled across resources. Each portal’s native preprocessing was retained; comparison was performed only regarding the direction of the resource-specific results. Because normal and tumor samples were unpaired and could differ in cellular composition, the pan-cancer analysis served only as a prioritization screen. Given the promising results for ovarian serous carcinoma (see Section 3.1), the subsequent phases of the study focused on this tumor type.

### 2.2. Assessing the Impact of TFAP2E Expression on Clinical Features Alongside Data Acquisition for Consecutive Analyses

The University of Alabama at Birmingham Cancer Data Analysis Portal (UALCAN) enabled the examination of the relationship between *TFAP2E* expression patterns and clinical parameters in the ovarian serous carcinoma cohort from the Cancer Genome Atlas (TCGA-OV). Analysis included tumor stage, histological grade, patient age, race, and *TP53* mutation status. Transcriptomic data of TCGA-OV patients for downstream analyses were retrieved as raw counts (Spliced Transcripts Alignment to a Reference protocol; STAR) and normalized values (transcripts per million; TPM) from the Genomic Data Commons (GDC) repository [18] via the GDCquery() function of the TCGAbiolinks v2.34.1 R-package. Corresponding clinical annotations were sourced from both the GDC and the TCGA Clinical Data Resource (TCGA-CDR) [19]. Initial survival analyses included 420 expression-matched cases. The overlap of endpoint-derived assignments comprised 391 cases and defined the transcriptomic analysis cohort; 380 had complete age, stage, and grade data. Availability of samples depending on the analysis is summarized in Supplementary File S1.

To examine genomic and epigenetic correlates of *TFAP2E* expression, methylation and copy number data for TCGA-OV patients were acquired from GDC. The HumanMethylation27 analysis included both promoter-proximal probes annotated to *TFAP2E* (cg11835197, TSS1500; cg26372517, 5′UTR/first exon). A position-based audit found no other 27 K probe within ±10 kb of the annotated transcription start site, and the mean beta value of the two available probes was used as a promoter composite. The tumor–normal methylation comparison used a separate assay-specific population of 582 tumors and 12 available solid-tissue normal samples, without requiring matched expression or survival data; methylation–expression analyses included 382 matched tumors. The HumanMethylation450 platform included eight promoter-proximal probes, but only nine tumors had matched expression data; this platform was therefore used only to assess probe coverage. Relative GISTIC2 copy number values with thresholded calls were available for 387 matched tumors. ASCAT3 absolute copy number was analyzed in 372 tumors as a sensitivity check. Spearman correlations and Wilcoxon tests were computed with R stats. Probe-level *p*-values were adjusted using the Benjamini–Hochberg method, whereas associations between methylation and copy number were assessed by partial Spearman correlations.

### 2.3. Survival Analysis and Assessment of Overlap Between Stratified Groups

*TFAP2E* expression cutpoints for the exploratory stratification were obtained with the “Cutp” method in EvaluateCutpoints [20]. In TCGA-OV, overall survival (OS), disease-specific survival (DSS), and progression-free interval (PFI) yielded a common TPM cutpoint of 1.9318, whereas disease-free interval (DFI) yielded 2.0486 and was not used to define the downstream groups. The common cutpoint produced patient subgroups representing lower and higher *TFAP2E* expression (294 and 97 individuals, respectively). A second exploratory cutpoint (0.6075) was estimated within the lower stratum to ultimately obtain “low”, “medium”, and “high” subgroups, each representing at least 20% of the cohort. Because both thresholds used survival information, these labels were retained as secondary exploratory strata rather than transferable clinical cutoffs. Kaplan–Meier plots were visualized using ggsurvplot() from survminer v0.5.0. Cox proportional hazards models were fitted using coxph() from survival v3.8-3, the proportional hazards assumption was assessed with Schoenfeld residuals using cox.zph(), and forest plots were generated with forest_model() from forestmodel v0.6.2. Overlap among OS-, DSS-, and PFI-derived assignments was examined using UpSetR v1.4.0.

Clinically adjusted TCGA-OV analyses were restricted to cases with complete covariate data and included age, stage, and histological grade. *TFAP2E* was modeled continuously per standard deviation and using the original and outcome-independent group definitions. The latter included an upper-quartile split, a two-component Gaussian mixture based on expression, and quartile-based three-group definitions. Global likelihood-ratio tests assessed the contribution of the *TFAP2E* groups, with Benjamini–Hochberg correction across the paired DSS and PFI analyses.

Sensitivity models added residual disease, somatic *BRCA1/2* status, consensus tumor purity, homologous recombination deficiency (HRD) score, ovarian mRNA subtype, primary therapy response, aneuploidy, and fraction genome altered. For DSS, Fine–Gray subdistribution hazard models and cumulative incidence curves were obtained using crr() and cuminc() from cmprsk v2.2-12 to assess competing non-cancer mortality. Competing risk analysis was restricted to DSS because non-cancer death is a coherent competing event for cancer-specific death. Discrimination beyond clinical-only models was evaluated using paired bootstrap differences in Harrell’s C-index, with 1000 replicates generated using boot() from boot v1.3-31.

### 2.4. Differential Expression Analysis

Differential expression analysis (DEA) was conducted using the limma-voom method in limma v3.62.2 [21]. Groups were defined solely by the upper quartile of *TFAP2E* expression, without survival-based cutpoint selection. The workflow included calcNormFactors() normalization, retention of transcripts with at least 5 counts per million in at least one library, and variance modeling with voom(). Weighted least-squares models were fitted with lmFit(), and log2 fold changes (log2FC) for the upper-quartile group relative to the remaining cases were estimated using makeContrasts(). Empirical Bayes moderation was applied before extracting results with topTable(). Differentially expressed genes (DEGs) were defined by a Benjamini–Hochberg adjusted *p*-value (FDR) < 0.05 and |log2FC| > 0.58. The global differential expression landscape was visualized with EnhancedVolcano v1.24.

### 2.5. Weighted Gene Correlation Network Analysis

Weighted gene correlation network analysis (WGCNA) [22] was implemented within the R environment to identify functional modules within the collection of genes previously associated with *TFAP2E* expression (obtained from our previous research [23]). The input gene collection was assembled without using patient survival information. Network construction employed a signed hybrid approach with a soft-thresholding power of 6, achieving a scale-free topology fit of over 0.85. Module identification used hierarchical clustering with the average linkage method via the hclust() function. The cutreeDynamic() algorithm identified modules with parameters set to require a minimum of 30 genes per module and moderate splitting sensitivity. Module eigengenes were tested against continuous *TFAP2E* expression using Spearman correlation, with BH correction across module tests. The pink module was selected by the strongest absolute association. Expression patterns across modules were visualized using heatmaps generated with heatmap.2() function from the gplots v3.1.1 R-package, employing Pearson’s distance metric for row clustering and the complete agglomeration method.

The network was reconstructed under fifteen alternative settings that varied soft-threshold power, deepSplit parameter, minimum module size, merge cut height, network type, Pearson versus biweight midcorrelation, and log2(TPM + 1) transformation. For each setting, the module best matching the reference pink module was evaluated by Jaccard overlap and retention of its fixed subsets. The association with continuous *TFAP2E* expression was assessed by Spearman correlation. Preservation of the reference module in GSE32062 was tested with WGCNA::modulePreservation() using 200 permutations. As an outcome-independent sensitivity check, gene-wise median absolute deviation (MAD) was calculated: the top 15,000 genes were tested for overlap with the pink module and the intersection-derived gene set using one-sided Fisher tests.

### 2.6. Network, Intersection, and Enrichment Analyses

The 933-gene pink module was analyzed in Metascape with its integrated Molecular Complex Detection (MCODE) algorithm [24] to identify densely connected network components. The intersection between the 874 genes identified by limma-voom and the 933 genes in the WGCNA pink module was visualized with draw.pairwise.venn() from VennDiagram v1.7.3. The resulting 82 genes were visualized with heatmap.2() from gplots v3.1.1 using the same clustering settings as in Section 2.5. The interactions within the intersection-derived set were assessed with GeneMANIA [25] without adding resultant genes.

To assess specificity while controlling for multiple testing, overrepresentation analysis was performed using clusterProfiler v4.14.6, ReactomePA v1.50.0, and msigdbr v24.1.0. Primary analyses tested each subset against the preceding set in the selection sequence: from the WGCNA input, through the pink module and the intersection-derived set, to the consensus subset identified in both cohorts. The two smaller subsets were additionally tested against WGCNA input genes. GO Biological Process, Reactome, and MSigDB Hallmark reported the input and mapped counts, gene and background ratios, fold enrichment, nominal *p*-values, and database-specific BH FDR.

For a complementary promoter analysis, one promoter per mapped gene (hg38; −1000/+200 bp relative to the transcription start site) was scanned with monaLisa v1.12.0 using GC- and k_mer-adjusted backgrounds. The global scan included 879 JASPAR 2024 CORE vertebrate models, with BH correction across 2529 evaluable tests. The AP-2 family sensitivity included JASPAR tests and additional evaluation using the HOCOMOCO v11, with BH correction across all 33 family tests. JASPAR provided models for *TFAP2A/B/C/E* (only one for *TFAP2E*), while HOCOMOCO v11 was the only source for *TFAP2D* [26,27]. Motif enrichment was interpreted as evidence of sequence compatibility, not transcription factor occupancy or regulation.

### 2.7. Reconsidering Stratification of Patients Alongside the Assessment of Immune Subtypes and the Stemness Index

Considering the WGCNA results and the expression profiles of the original *TFAP2E* groups, the lower expression stratum was further divided using the exploratory second cutpoint described in Section 2.3, while the “high” group was left unchanged. The resulting “low”, “medium”, and “high” groups were retained for secondary exploratory analyses; they were not interpreted as a clinical classifier. Sensitivity was assessed with outcome-independent quartile groups and with continuous *TFAP2E* relationships to the pink module eigengene and PCA coordinates. Survival analyses used survminer v0.5.0 and forestmodel v0.6.2 as described above. The 82-gene expression profile was visualized with heatmap.2(). Immune subtypes were assigned using the ImmuneMW.rda resource and TCGAanalyze_ImmuneSubtypes(), while mRNAsi and EREG-mRNAsi values were obtained from published data [28]. Immune subtype distributions were displayed with ggplot2 v3.5.1, and stemness scores with beanplot v1.2; Sidak’s test was used for the reported group comparisons. Immune and stemness results were treated as exploratory.

### 2.8. Dimensionality Reduction and Sample Clustering Analysis

Principal component analysis (PCA) [29] was implemented in the R environment to reduce dimensionality and visualize sample distribution patterns, using intersection-derived genes as input variables. The analytical procedure incorporated data scaling and centering as preprocessing steps to ensure standardized variance contributions. The PCA methodology was implemented using the FactoMineR v2.4 R-package, with visualization facilitated through the factoextra v1.0.7 R-package. Spatial discrimination between “*TFAP2E*-high”, “*TFAP2E*-medium”, and “*TFAP2E*-low” groups of patients was visualized across the first two principal components (PC1/Dim1 and PC2/Dim2). Data inferred from survival analysis, immune subtyping, and stemness scoring were manually added to the PCA plot using Inkscape v1.2 [30]. Additional PCA sensitivity analyses used stats::prcomp() on centered and scaled expression data, Pearson and Spearman correlations with continuous *TFAP2E* expression, and a Euclidean pseudo-F test of group separation across all intersection-derived genes with 1999 label permutations.

### 2.9. Cross-Cohort Analyses

The effects of *TFAP2E* on patient survival, expression of intersection-derived genes, immune subtyping, and the stemness index were verified in an independent cohort of patients. The GSE32062 cohort from the Gene Expression Omnibus (GEO) was selected because it includes progression-free survival (PFS) data in addition to OS. Its relatively large sample size of 260 patients also enabled stratification into three *TFAP2E* expression groups, each comprising at least 20% of the cohort, consistent with the approach used in TCGA-OV. At this stage, it was decided to set a cutpoint only for PFS, since events due to disease progression occur earlier than death [31]. EvaluateCutpoints, survminer, forestmodel, TCGAanalyze_ImmuneSubtypes(), and heatmap.2() were used as described above. GSE32062 mRNAsi was calculated with TCGAanalyze_Stemness() using SC_PCBC_stemSig.rda; EREG-mRNAsi could not be calculated because the cohort lacked the required data.

Regarding clinical information, age was unavailable in GSE32062, whereas all patients were annotated as having received platinum and taxane treatment. Clinically adjusted analyses used complete cases and included stage, grade, and cytoreductive-surgery status. *TFAP2E* was evaluated continuously per standard deviation and using the outcome-independent median split, quartile-based grouping, and expression-only Gaussian mixture models. A three-component Gaussian mixture was included as a secondary sensitivity analysis. Cox model fitting, proportional-hazards diagnostics, and C-index comparisons followed the procedures described in Section 2.3. TCGA PFI and GSE32062 PFS estimates were combined in an inverse variance common-effect analysis; a DerSimonian and Laird random-effects model served as a sensitivity analysis. For each cohort, a maximally selected log-rank scan evaluated candidate group fractions of 10–50% using 100,000 outcome-label permutations. The corrected *p*-values were then combined using Fisher’s method. The permutation analysis and Fisher combination were implemented using sample.int() to generate outcome-label permutations and pchisq() to calculate Fisher’s combined *p*-value.

A subset of intersection-derived genes (denoted as “consensus genes”) with verified expression profile changes (based on *TFAP2E* groups) was analyzed for their genomic location and regulation. Available GTRD binding data for AP-2α and AP-2γ and HOCOMOCO position-weight matrices [32] were used only as family-level contextual evidence; they do not demonstrate AP-2ε genomic occupancy. MACRO-APE [33] was used to compare AP-2 binding profiles via the Jaccard index. Pairwise Spearman correlations among the five AP-2 family genes were calculated separately in TCGA-OV and GSE32062, with BH correction across the ten gene pairs within each cohort. In TCGA-OV, expression of *TFAP2A–D* was additionally compared between the original *TFAP2E* groups using rank-based tests, with BH correction across the four genes. These analyses assess coexpression only and cannot demonstrate redundancy, compensation, dimerization, or AP-2 activity.

### 2.10. Cancer Hallmark Enrichment, Protein-Level Characterization, and Multivariable Analysis

Cancer-related processes were assessed with CancerHallmarks [34] for the pink module identified by WGCNA (933 genes) and a subset of this module, obtained by intersecting it with limma-voom results (82 genes). Intersection-derived genes were also investigated for their protein expression patterns using immunohistochemical (IHC) specimens obtained from the Human Protein Atlas (HPA) [35]. All 82 entries were screened. Usable IHC data were available for 51 of them, represented by 52 entries because *STX16-NPEPL1* was assessed through its two component proteins. The same antibodies were used to examine both normal ovarian tissues and tumor samples, obtained from the “Tissue” and “Pathology” atlases, respectively. Reported data include the antibody and patient identifiers, staining category, assessed cell type, cellular localization, and HPA’s reliability category. The comparisons were descriptive and semiquantitative, and no custom IHC score was calculated.

Multivariable Cox proportional hazards models were fitted separately for each survival endpoint in the TCGA-OV cohort using intersection-derived gene expression, with the coxph() function from the survival v3.8-3 R package. Patients were stratified for each gene using the optimal expression cutoff, and the resulting hazard ratios with 95% confidence intervals were visualized as summary forest plots using the forest() function from the metafor v4.9-4 R package. Clinically adjusted continuous Cox models were additionally fitted for each intersection-derived gene using coxph() from survival v3.8-3, for DSS and PFI in TCGA-OV and PFS in GSE32062. Effect directions were compared across cohorts, and Wald *p*-values were BH-adjusted across the genes tested within each cohort and endpoint.

Protein-coding genes significant in both univariable and expression-based multivariable Cox analyses in at least two endpoints were highlighted. The resulting seven genes (*AQP6*, *RGL3*, *KCNQ4*, *COL11A2*, *EPOR*, *DSCAML1*, and *LRRC37A2*) were further evaluated in joint Cox models. Each gene entered the model as a separate continuous covariate. Core TCGA-OV models included all seven candidates plus age, stage, and grade. Sensitivity models added *BRCA1/2* status, residual disease, tumor purity, and treatment response. *MUC16* and *WFDC2* expression were also added as a proxy analysis for CA125 and HE4. All seven genes represented in GSE32062 were examined in stage-, grade-, and surgery-adjusted PFS models. Global likelihood-ratio tests assessed joint contribution, and BH correction was applied to *p*-values for individual candidate coefficients within each model.

## 3. Results

### 3.1. Preliminary Pan-Cancer Context and TFAP2E Survival Groups

Across the three queried resources, ovarian cancer showed the clearest concordance of lower tumor *TFAP2E* expression in microarray and RNA-Seq data (Supplementary Figure S1A–C). Routine clinicopathological variables were not associated with *TFAP2E* expression in TCGA-OV (Supplementary Figure S1D). The expanded analysis found no association with stage, grade, residual disease, primary therapy response, *BRCA1/2* status, or mRNA subtype, and no correlation between continuous *TFAP2E* expression and tumor purity. This prompted a more comprehensive analysis with transcriptomic profiling.

**Figure 1:**
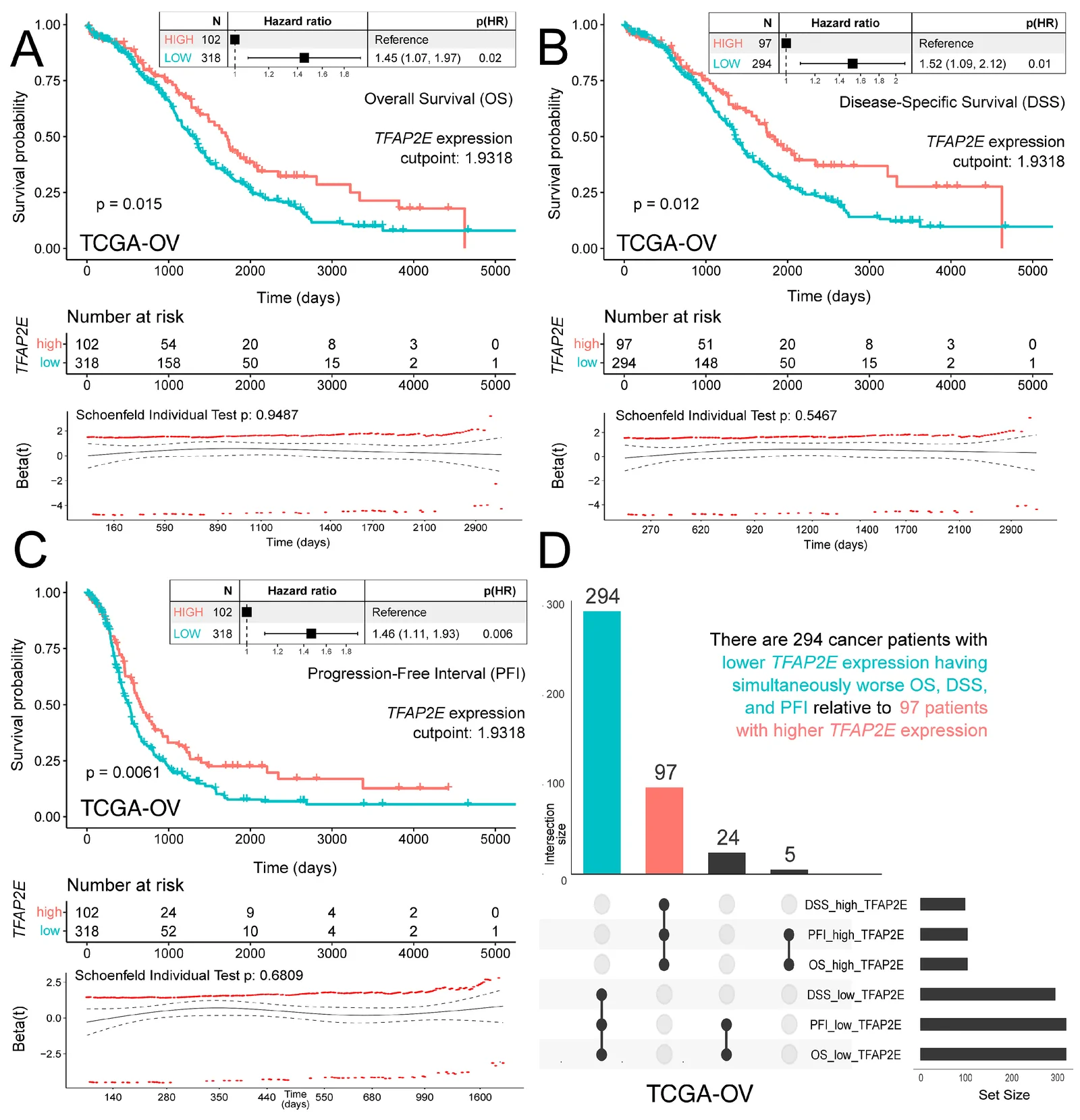
*TFAP2E* expression stratifies the survival outcomes in TCGA-OV and defines exploratory survival groups. Kaplan–Meier plots are shown for (**A**) OS, (**B**) DSS, and (**C**) PFI, along with (**D**) the overlap used to define the *TFAP2E*-low and *TFAP2E*-high groups.

*TFAP2E* was investigated for its influence on survival of TCGA-OV patients, revealing that its higher expression was associated with favorable OS, DSS, PFI, and DFI. The first three endpoints shared the 1.9318 TPM cutpoint (Figure 1A–C), whereas DFI indicated a different threshold (2.0486 TPM) and was excluded from the downstream group definition. The overlap of OS-, DSS-, and PFI-derived assignments (Figure 1D) defined groups with relatively lower and higher *TFAP2E* expression (294 and 97 cases, respectively). Henceforth, the first group is denoted as “*TFAP2E*-low” while the second as “*TFAP2E*-high”. These survival-derived groups were retained for secondary exploratory analyses, whereas DEG/WGCNA discovery used outcome-independent expression definitions.

Neither of the two promoter-proximal 27 K probes nor their composite was associated with *TFAP2E* expression (rho = −0.013, *p* = 0.797). Composite methylation was lower in tumors than in solid-tissue normal samples (median 0.296 versus 0.477; *p* = 1.81 × 10^−7^). GISTIC2 relative copy number showed a modest positive association with expression (rho = 0.193, *p* = 1.37 × 10^−4^). This association remained after adjustment for methylation (rho = 0.184, *p* = 3.19 × 10^−4^), but the ASCAT3 analysis showed a weaker association (rho = 0.094, *p* = 0.069). Thus, relative copy number may contribute modestly to inter-tumor variation, whereas the assayed methylation loci did not; neither result establishes causality (Supplementary File S1).

### 3.2. Identification of TFAP2E-Associated Genes by Differential Expression and WGCNA

**Figure 2:**
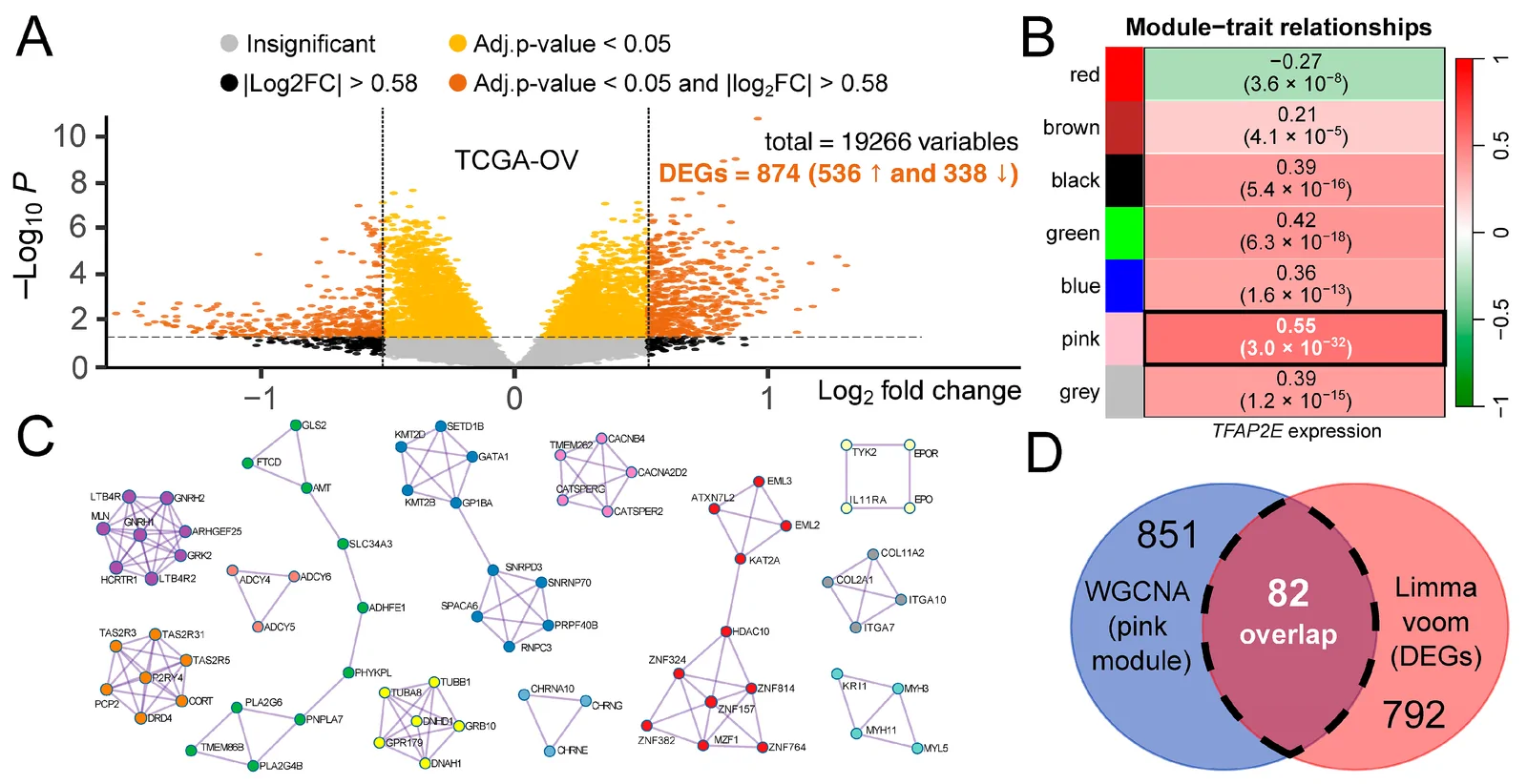
Differential expression, WGCNA, and network analysis. (**A**) Volcano plot highlighting DEGs. (**B**) Module–trait relationships. (**C**) Compact MCODE components from the pink module. (**D**) Overlap between DEGs and the pink module. Full differential expression tables are summarized in Supplementary File S2. Interactive networks are available in Supplementary Files S3 and S4. The pink module heatmap, static network panels, robustness, MAD, and enrichment results are provided in Supplementary File S5.

The upper-quartile definition closely matched the original survival-derived split (Supplementary File S2). Using the expression-only definition, limma-voom identified 874 genes at BH FDR < 0.05 and |log2FC| > 0.58 (Figure 2A). Immunoglobulin genes were among the most differentially expressed, potentially reflecting immune-cell composition and tumor biology; the full results and top table are provided in Supplementary File S2.

WGCNA identified coexpression modules associated with continuous *TFAP2E* expression (Figure 2B). The 933-gene pink module showed the strongest positive relationship with *TFAP2E* expression (Spearman rho = 0.550, *p* = 3.03 × 10^−32^; FDR = 2.12 × 10^−31^). MCODE identified densely connected components within the pink module (Figure 2C; Supplementary File S3). Afterwards, an overlap of the 874 differentially expressed genes with the pink module produced the intersection-derived set encompassing 82 genes (Figure 2D). Network properties generated from these overlapping genes confirmed their coexpression pattern (Supplementary File S4).

Across 15 alternative WGCNA settings, matched modules retained 84.1–100% of the pink module and 86.6–100% of the intersection-derived set, with a median Jaccard index of 0.639. Their eigengenes remained associated with continuous *TFAP2E* expression (rho = 0.547–0.553; FDR ≤ 6.50 × 10^−32^). The outcome-independent MAD screen retained 524/933 module genes (56.2%; fold enrichment = 1.52; *p* = 3.45 × 10^−33^) and 62/82 intersection genes (75.6%; fold enrichment = 2.04; *p* = 1.27 × 10^−12^). Complete robustness and MAD tables are provided in Supplementary File S5.

### 3.3. Cross-Cohort Survival and Expression Patterns

**Figure 3:**
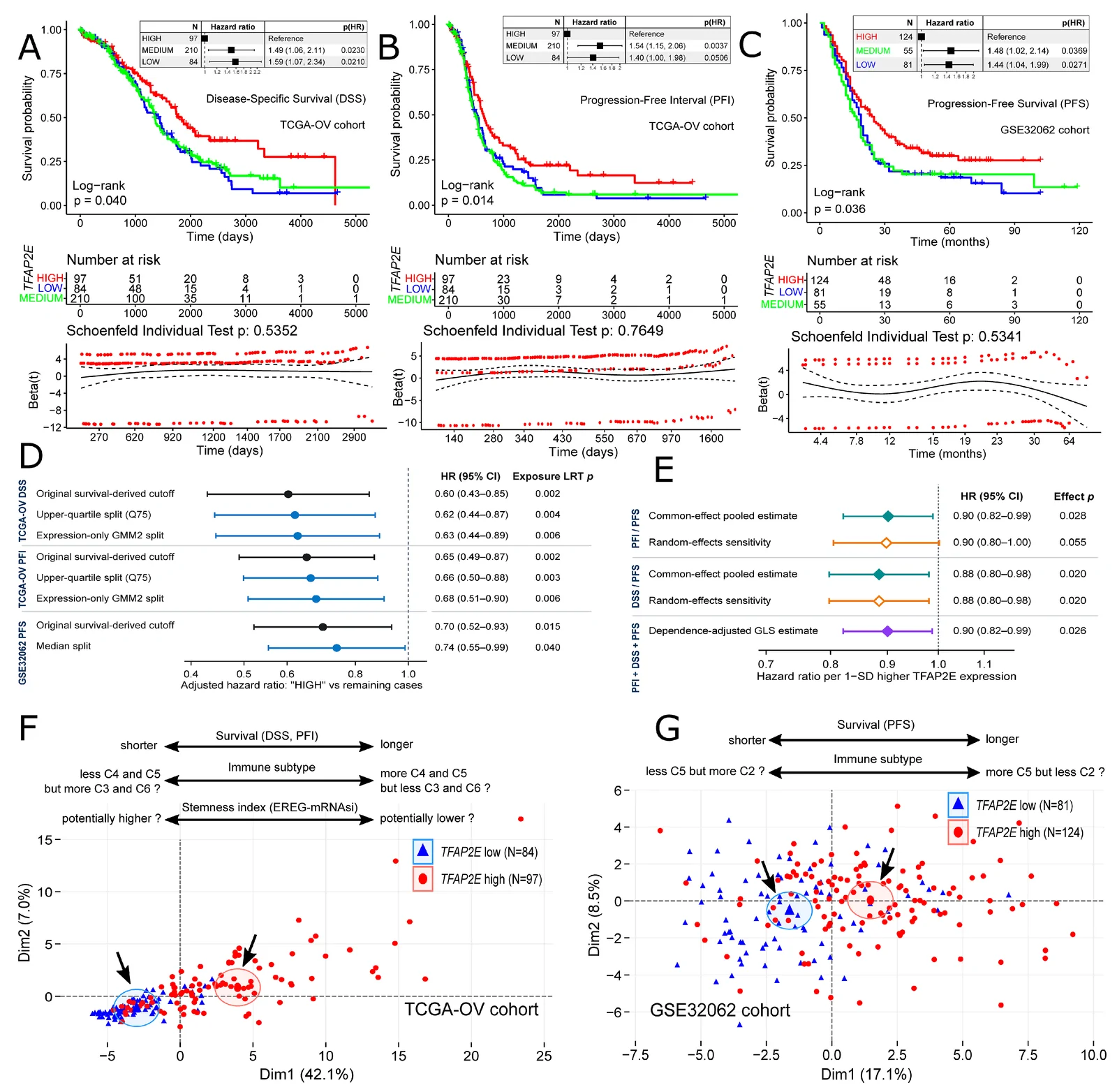
Cross-cohort survival and expression comparison of *TFAP2E* groups. Kaplan–Meier plots for (**A**) TCGA-OV DSS, (**B**) TCGA-OV PFI, and (**C**) GSE32062 PFS. (**D**) Clinically adjusted estimates for the original grouping and outcome-independent alternatives: the upper quartile, a two-component Gaussian mixture (GMM2), and the median split. TCGA-OV- and GSE32062-adjusted models used 380 and 260 complete cases, respectively. Circles distinguish original (black) and outcome-independent (blue) groupings. (**E**) Cross-cohort continuous estimates with effect *p*-values, using endpoint-specific complete cases (TCGA-OV PFI: 408; TCGA-OV DSS: 380; GSE32062 PFS: 260). Diamonds indicate common-effect (teal), random-effects (orange), and dependence-adjusted (purple) estimates. (**F**,**G**) PCA of TCGA-OV and GSE32062 based on the expression of intersection-derived genes. Supplementary File S6 contains the complete information regarding grouped, continuous, pooled, and competing risk analyses.

Given the disproportion in group size and a noticeable gradual change in the expression profile of patients depending on the *TFAP2E* level (pink module heatmap in Supplementary File S5), it was decided to isolate a “medium” group subset from the “low” group (the “high” group remained unchanged), taking into account that each group represented at least 20% of the entire TCGA-OV cohort. Reanalysis of survival endpoints by DSS (Figure 3A) and PFI (Figure 3B) indicates separation of the *TFAP2E*-high group from the other two groups. In covariate-complete cases, both the original and quartile-based groups remained associated with DSS and PFI (maximum global *p* = 0.0150). The adjusted high-vs-remaining comparisons were also consistent (Figure 3D).

GSE32062 cohort was used to assess whether the survival association and expression pattern were present outside TCGA-OV. The three-group comparison was associated with PFS (Figure 3C) and remained such after adjustment for stage, grade, and cytoreductive-surgery status (global *p* = 0.0493). The adjusted high-vs-remaining comparison was consistent with that of TCGA-OV (Figure 3D). Continuous *TFAP2E* was associated with GSE32062 PFS (HR per SD = 0.840, 95% CI 0.722–0.977, *p* = 0.0235). Cross-cohort estimates were directionally favorable in the common- and random-effects models, as well as in the dependence-adjusted model (Figure 3E). Complete three-group, PH-stratified, competing risk, discrimination, and cutoff sensitivity results for both cohorts are provided in Supplementary File S6.

Spatial analysis using the expression of intersection-derived genes indicates that the “*TFAP2E*-high” and “*TFAP2E*-low” groups are separated along principal component dimensions (Figure 3F,G), with the “*TFAP2E*-medium” group showing a transitional profile between the other two groups (Supplementary File S6). PC1 correlated with continuous *TFAP2E* expression in both cohorts (TCGA-OV: rho = 0.582, *p* = 8.45 × 10^−37^; GSE32062: rho = 0.477, *p* = 3.37 × 10^−16^). Permutation tests of the gene expression profiles supported group separation under both the original and outcome-independent definitions in each cohort (*p* = 0.0005 for all tested definitions). Exploratory immune subtyping and stemness comparison were also performed in both cohorts (Supplementary Figure S2). Immune subtype distributions varied modestly, with a potentially higher C5 proportion in the *TFAP2E*-high groups. Neither mRNAsi nor EREG-mRNAsi differed significantly among the three groups; the low-vs-high comparison for EREG-mRNAsi in TCGA-OV showed a borderline trend (*p* = 0.0568). Data inferred from survival analysis, immune subtyping, and stemness scoring were manually added to the PCA plots.

### 3.4. Functional Context and Expression Concordance of TFAP2E-Associated Genes

**Figure 4:**
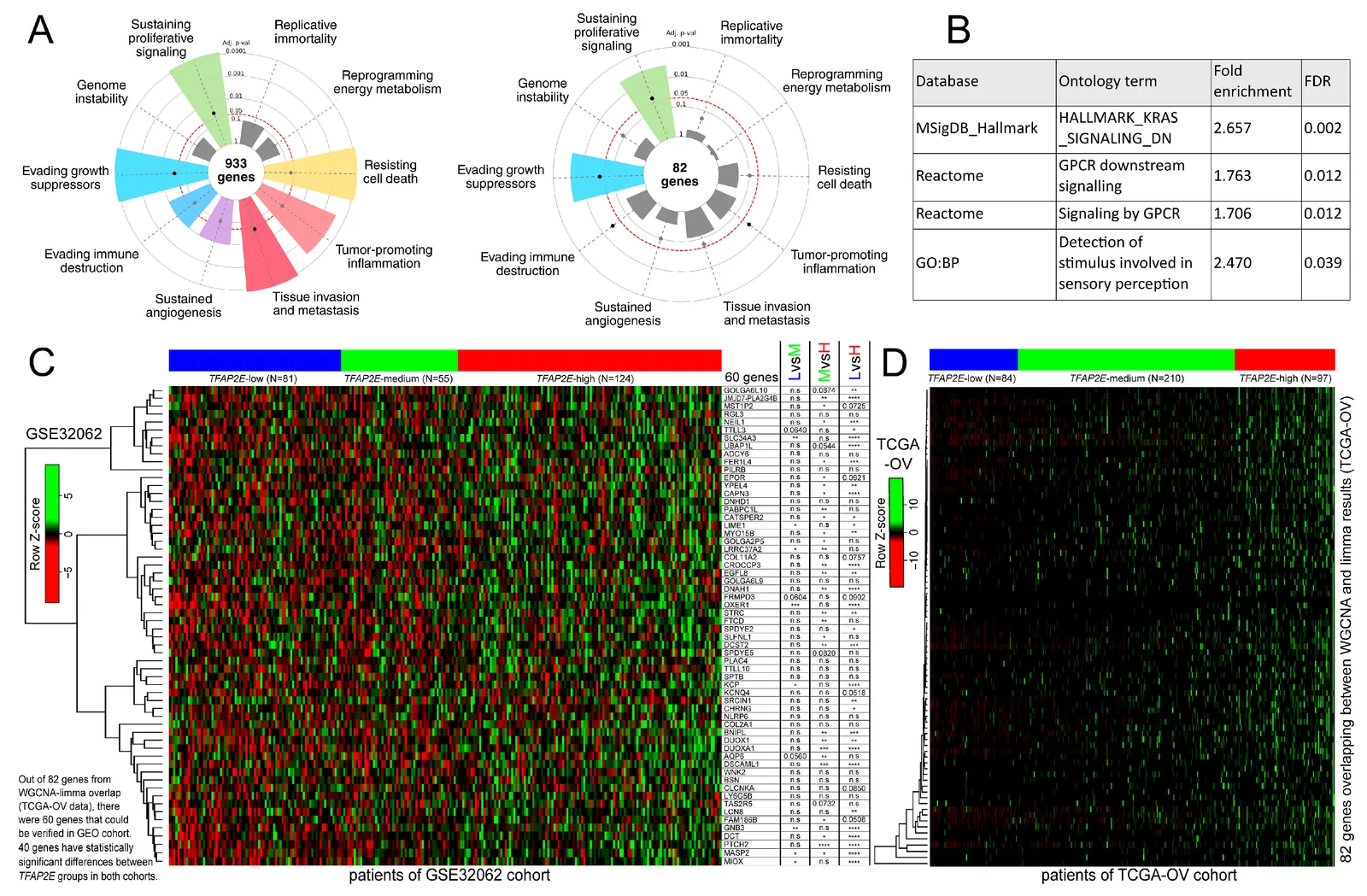
Functional context of *TFAP2E*-related gene sets and cross-cohort concordance. (**A**) CancerHallmarks summaries for the pink module and intersection-derived set. (**B**) Enrichment results for the pink module using genes included in WGCNA as the background. (**C**) GSE32062 expression heatmap for 60 of 82 intersection-derived genes available on the microarray. (**D**) TCGA-OV heatmap for the entire intersection-derived set. The bar annotations for heatmaps retain the original exploratory survival strata (260 and 391 cases, respectively). Complete enrichment tables are provided in Supplementary File S5, and detailed cross-cohort gene models are provided in Supplementary File S7. L—“*TFAP2E*-low”; M—“*TFAP2E*-medium”; H—“*TFAP2E*-high”. * *p* < 0.05, ** *p* < 0.01, *** *p* < 0.001, **** *p* < 0.0001, n.s., not significant.

Cancer hallmark analysis of the intersection-derived set indicated enrichment of sustaining proliferative signaling and evading growth suppressors. However, other hallmarks, such as resistance to cell death and tumor-promoting inflammation, were represented when extending the scope to the entire pink module, from which intersecting genes were extracted (Figure 4A). Complementary enrichment analysis identified GPCR signaling and sensory stimulus detection in the pink module, along with a reference signature of genes downregulated following KRAS activation (Figure 4B).

Expression comparisons among the *TFAP2E* groups identified 40 genes with significant differences in both GSE32062 (Figure 4C) and TCGA-OV (Figure 4D), hereafter termed “consensus genes”. These were further analyzed for their genomic location to infer whether they can be bound by the AP-2 family. This step was performed using data for AP-2α and AP-2γ due to the lack of appropriate information for AP-2ε (details in Section 2.9) and given the resemblance of the Jaccard index for all AP-2 factors except AP-2δ (Supplementary File S7). Twenty-one of the consensus genes had AP-2α and/or AP-2γ binding annotations in GTRD. However, AP-2α/γ and motif similarity information provide only family-level context, not inference of chromatin occupancy specific to AP-2ε. *TFAP2E* correlated modestly with *TFAP2C* in TCGA-OV (rho = 0.226; FDR = 6.37 × 10^−5^); none of the correlations between *TFAP2E* and the other AP-2 family members were significant in GSE32062. The promoter scan yielded no globally significant motif or AP-2 family result (Supplementary File S5). Cross-cohort gene models, sensitivity analyses, and AP-2 genomic context are detailed in Supplementary File S7.

### 3.5. Protein-Level Characterization and Prognostic Significance of Candidate Genes

**Figure 5:**
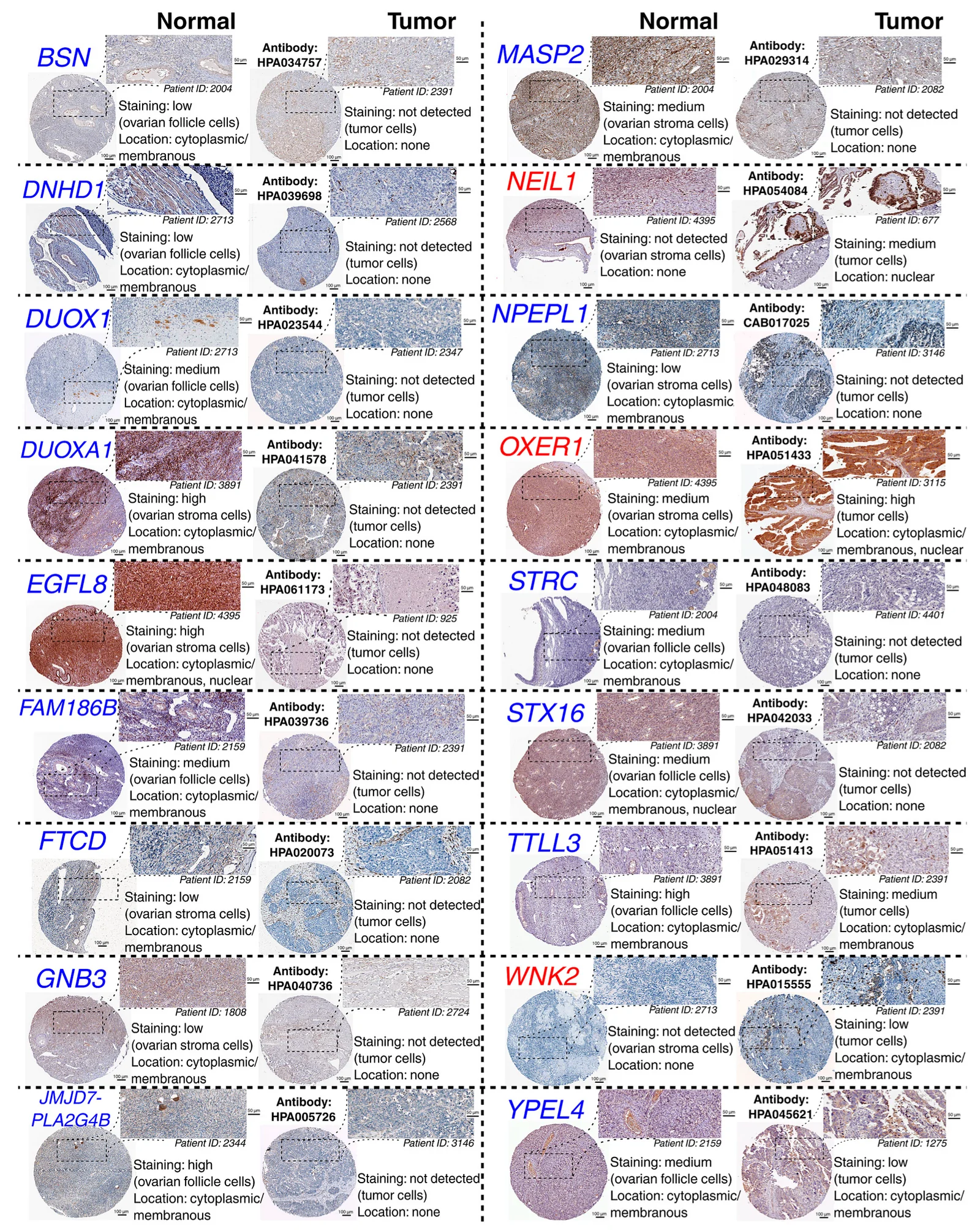
Representative immunostaining patterns for the intersection-derived set. Normal and ovarian cancer specimens are antibody-matched. Blue font denotes higher staining in the representative normal specimen, and red font indicates higher staining in the representative tumor specimen. The intersection-derived set coverage, antibody and patient identifiers, localization, staining categories, and reliability annotations are provided in Supplementary File S8.

IHC investigation of the intersection-derived set uncovered staining differences between normal and tumor specimens for 17 genes (*BSN*, *DNHD1*, *DUOX1*, *DUOXA1*, *EGFL8*, *FAM186B*, *FTCD*, *GNB3*, *JMJD7-PLA2G4B*, *MASP2*, *NEIL1*, *OXER1*, *STRC*, *STX16-NPEPL1*, *TTLL3*, *WNK2*, *YPEL4*), with *STX16-NPEPL1* represented by separate *STX16* and *NPEPL1* staining data. Except for *NEIL1*, *OXER1*, and *WNK2*, the remaining genes with distinct patterns showed decreased staining in the tumor compared with normal ovarian tissue (Figure 5). Information regarding antibody, patient, localization, staining, and reliability status is provided in Supplementary File S8.

The intersection-derived set was also subjected to expression-based multivariable Cox models, revealing genes with potential prognostic influence after mutual adjustment for the other genes included in the model across OS, DSS, DFI, and PFI (forest plots in Supplementary File S7). Protein-coding genes significant in both univariable and expression-based multivariable analyses in at least two endpoints included *AQP6*, *RGL3*, *KCNQ4*, *COL11A2*, *EPOR*, *DSCAML1*, and *LRRC37A2*. The joint contribution of this gene set to the age-, stage-, and grade-adjusted DSS model was significant in TCGA-OV (global *p* = 0.0462), and remained such after adding somatic *BRCA1/2* status (global *p* = 0.0329). This association was also retained in the broader model including residual disease, purity, *BRCA1/2* status, as well as *MUC16* and *WFDC2* expression as available proxies for the CA125 and HE4 context (global *p* = 0.00802). All seven genes were available in GSE32062, but their joint contribution to the PFS model adjusted for stage, grade, and cytoreductive-surgery status was not significant (global *p* = 0.251). Candidates highlighted by the complementary survival and protein-level analyses remain exploratory. The complete endpoint and sensitivity matrix is provided in Supplementary File S7.

## 4. Discussion

The AP-2 family is an essential group of transcription factors that regulate gene expression during embryonic development and differentiation. These TFs play imperative roles in the initiation and progression of cancer, with prognostic consequences for oncological patients [36]. The function of AP-2ε has not been studied in many cancer types or is incompletely understood in others [36,37,38]. Across the three queried pan-cancer resources, ovarian carcinoma showed the clearest concordance of lower *TFAP2E* expression in tumor relative to corresponding normal tissue. This finding motivated an exploratory workflow utilizing survival, transcriptomic, and clinical data, with cross-cohort comparison and immunohistochemistry support. These analyses identify a transcriptomic pattern associated with *TFAP2E* expression; they do not establish that AP-2ε directly regulates the observed genes or phenotypes. The available literature is likewise context-dependent: *TFAP2E* methylation has been linked to colorectal cancer chemoresistance [14], its lower expression to adverse outcomes in oral squamous cell carcinoma [39], and higher expression to melanoma progression [40].

Although *TFAP2E* was not associated with routine TCGA-OV clinicopathological variables, its cross-platform downregulation and survival association suggest that *TFAP2E*-linked transcriptional states may capture differences not fully reflected by standard clinical assessment. Other tumors show context-dependent associations with clinical features [41,42]. *TFAP2E* methylation has also been associated with gastric cancer differentiation [43] and colorectal cancer staging [44]. Our results support a clinically adjusted association between higher *TFAP2E* expression and favorable survival, but do not establish AP-2ε as a functional tumor suppressor in ovarian serous carcinoma. Protective associations have been reported in oral squamous cell carcinoma [39], bladder cancer [42], and neuroblastoma [13].

The pink module and intersection-derived set defined a transcriptomic pattern associated with *TFAP2E* expression, with pink-module enrichment providing biological context. GPCR signaling linked to the pink module contributes to cytoskeletal remodeling and migration in high-grade serous ovarian cancer cells through lysophosphatidic acid receptors [45]. The enrichment for sensory stimulus detection has an indirect connection to the role of *TFAP2E* in maintaining vomeronasal sensory neuron identity [46]. In high-grade serous ovarian cancer cells, increased wild-type *KRAS* expression has been shown to promote resistance to cisplatin and carboplatin [47]. In KRAS-driven pancreatic cancer, squalene metabolism regulates *TFAP2E* expression and mitochondrial biogenesis dependent on PGC-1α [48]. These findings identify transcriptomic associations for further testing but not direct AP-2ε targets or the *TFAP2E*-specific mechanism. Furthermore, the absence of AP-2 family motif enrichment or a family-wide expression pattern argues against interpreting the pattern as evidence of AP-2ε-mediated regulation.

The predominance of immunoglobulin DEGs suggests an immune composition signal. Lung cancer showed *TFAP2E* correlations with several immune cell types [41], and bladder cancer showed associations with B cells and macrophages [42]. Across TCGA-OV and GSE32062, immune subtyping differences were modest and cohort-dependent, with only a small C5 subtype enrichment in higher-expression groups. C5 denotes the immunologically quiet category in the pan-cancer immune classification [49]. Its modest enrichment in the *TFAP2E*-high groups may reflect differences in immune composition, but does not establish AP-2ε-mediated immune activation or exhaustion.

Because transcription factors can influence cancer stemness [50], mRNAsi and EREG-mRNAsi were also examined; neither differed significantly among the three groups, although the low-vs-high comparison in TCGA-OV suggested higher EREG-mRNAsi in the *TFAP2E*-low group (*p* = 0.0568). Two promoter CpGs were unrelated to inter-tumor *TFAP2E* expression, and methylation was lower in tumor than in the corresponding normal tissue. Relative copy number showed a modest association, but neither assay established a mechanism of dysregulation. Prior work links the AP-2 family to context-specific epigenetic effects [51,52]. *TFAP2E* methylation has been linked to chemoresistance of both colorectal [14] and gastric cancer [43,53]. EMT and stemness are relevant to chemoresistant ovarian cancer [54,55], but generally their prognostic associations remain context-dependent [52,56,57], as do AP-2 family associations [36,58]. Our insignificant stemness results do not support a differentiation mechanism. Opposite *TFAP2E* associations in melanoma [40] and oral squamous cell carcinoma cells [39] further argue against a universal role.

IHC investigation of the intersection-derived set revealed normal-vs-tumor differences for 17 genes, among which only *NEIL1*, *OXER1*, and *WNK2* showed higher tumor staining. These three genes have been linked to adverse cancer phenotypes [59,60,61], but evidence from other tumors may not apply to ovarian serous carcinoma. The predominance of lower staining in cancer was consistent with the protective transcriptomic associations, but did not establish tumor-suppressive function. Reduced *FTCD*, *GNB3*, or YPEL family expression has been associated with aggressive tumors [62,63,64]. *YPEL4* has been linked to MAPK signaling [65], whereas *YPEL3* can induce senescence [66,67]. Reports of context-dependent *YPEL4* effects on proliferation [68], together with family-level observations [64,69], do not support universal tumor suppressor characteristics. *STRC* is a putative susceptibility gene in breast and ovarian cancer [70], but remains poorly described [70,71,72].

The seven highlighted genes (*AQP6*, *RGL3*, *KCNQ4*, *EPOR*, *COL11A2*, *DSCAML1*, *LRRC37A2*) have biological context supported by studies in ovarian cancer and other tumor types. *AQP6* has been examined in epithelial ovarian tumors, although its reported reduction in malignant tumors compared with benign ones was not statistically significant [73]. EPO/EPOR signaling has a more direct experimental connection to treatment response: prolonged erythropoietin exposure promoted a paclitaxel-resistant phenotype in ovarian cancer cells [74]. Higher *RGL3* expression has been associated with more favorable outcomes in breast cancer [75]. In breast cancer models, *KCNQ4* limited growth and invasion through AKT-related signaling [76]. *COL11A2* was part of a collagen expression pattern associated with poor survival in bladder cancer, providing extracellular matrix context [77]. *DSCAML1* was included in a prognostic model for stage IV colorectal cancer [78]. *LRRC37A2* was implicated in susceptibility to high-grade serous ovarian cancer in a transcriptome-wide study [79]. The joint association of these seven genes with DSS was supported in the age–stage–grade model and after adding *BRCA1/2* status, or in a broader model incorporating *MUC16* and *WFDC2* expression. Nevertheless, the PFS model was not significant in GSE32062, so these genes remain exploratory candidates requiring further research.

Several limitations should be acknowledged. The TCGA-OV and GSE32062 groups remain secondary exploratory strata, not calibrated cutoffs; primary DEG/WGCNA discovery used expression-only definitions. Nevertheless, it is important to note that continuous, permutation, DEA, WGCNA, and PCA robustness analyses supported the direction and downstream stability. Adjusted, stratified, and competing-risk models supported the group associations, but improved discrimination was not established. Detailed treatment and longitudinal sampling were unavailable. *MUC16* and *WFDC2* expression was examined only as an imperfect proxy for CA125 and HE4; they cannot establish prognostic value independent of serum concentrations, which were unavailable. All seven candidates were measured in GSE32062, but clinical comparability was limited by missing age information and by data on PFS rather than DSS/PFI. Methylation coverage was limited to two 27 K probes, nine expression-matched 450 K tumors, and twelve mostly unpaired normal samples. Bulk composition and immunostaining cannot establish AP-2ε abundance, *TFAP2E*-specific regulation, or tumor suppressive function. Direct binding requires perturbation and chromatin assays, and clinical assessment requires a prospective cohort. WGCNA sensitivity, external preservation, and MAD overlap support stability but not independence.

Nevertheless, the major strength of this study is the convergence of several complementary analytical layers. Such design supports the prioritization of *TFAP2E*-related genes for translational investigation in ovarian cancer. Limitations also define testable next steps. Future studies could examine *TFAP2E* overexpression or knockdown in ovarian serous carcinoma models with low or high endogenous expression. RNA-Seq could then test whether these perturbations alter the pink module profile and highlighted biological processes. AP-2ε-specific CUT&RUN or ChIP-Seq and promoter assays should then distinguish direct responsive loci from family motif compatibility. Because *TFAP2C* was the only family member with a modest coexpression relationship with *TFAP2E*, single-gene and combined perturbations could test compensation. These molecular experiments should be coupled to proliferation, migration, invasion, and responses to platinum and taxane treatment, followed by assessment in prospectively annotated cohorts. Finally, single-cell or deconvolution-based approaches could clarify whether the C5 pattern is related to AP-2ε. From a clinical perspective, *TFAP2E* and related gene sets should be assessed for prognostic value alongside standard clinicopathological variables and evaluated for utility in translational research.

## 5. Conclusions

To conclude, *TFAP2E* expression is associated with survival and a cross-cohort transcriptomic pattern in ovarian serous carcinoma. The *TFAP2E* survival association persisted after clinical adjustment and competing-risk sensitivity analysis. GSE32062 verification cohort supported concordant expression patterns and survival association. Cancer hallmark mapping, functional enrichment, motif analysis, and protein-level characterization provided biological context without establishing direct AP-2ε regulation or tumor-suppressive function. Immune-subtype and stemness findings remain exploratory. The highlighted *TFAP2E*-related genes (e.g., *AQP6*, *RGL3*, *KCNQ4*, *COL11A2*, *EPOR*, *DSCAML1*, and *LRRC37A2*) warrant further prognostic investigation. Overall, these findings provide a multilayered rationale for mechanistic and clinical evaluation of regulatory programs centered on *TFAP2E*/AP-2ε in ovarian serous carcinoma.

## Data Availability

The original contributions presented in this study are included in the article/Supplementary Material. Further inquiries can be directed to the corresponding author.

## Supplementary Materials

The following supporting information can be downloaded at https://www.mdpi.com/article/10.3390/cancers18193168/s1. Supplementary Figure S1. Pan-cancer expression profiling of *TFAP2E* and related clinicopathological context (Supplementary_Figure_S1.tif). Supplementary Figure S2. Exploratory immune subtype and stemness index comparisons (Supplementary_Figure_S2.tif). Supplementary File S1. *TFAP2E* methylation and copy-number analyses (Supplementary_File_S1.xlsx). Supplementary File S2. Differential expression results and robustness analyses (Supplementary_File_S2.xlsx). Supplementary File S3. Interconnection and MCODE clustering of the pink module at the protein level (Supplementary_File_S3.cys). Supplementary File S4. GeneMania’s assessment of network properties for the intersection-derived gene set (Supplementary_File_S4.cys). Supplementary File S5. WGCNA robustness, MAD analyses, enrichment, and AP-2 family analyses (Supplementary_File_S5.xlsx). Supplementary File S6. Survival, clinical adjustment, competing-risk, PCA, and cross-cohort preservation analyses (Supplementary_File_S6.xlsx). Supplementary File S7. Cross-cohort gene models, sensitivity analyses, and AP-2 genomic context (Supplementary_File_S7.xlsx). Supplementary File S8. Human Protein Atlas analysis of intersection-derived gene set with reliability audit (Supplementary_File_S8.xlsx).
